# Effect of Climate Factors on the Childhood Pneumonia in Papua New Guinea: A Time-Series Analysis

**DOI:** 10.3390/ijerph13020213

**Published:** 2016-02-15

**Authors:** Jinseob Kim, Jong-Hun Kim, Hae-Kwan Cheong, Ho Kim, Yasushi Honda, Mina Ha, Masahiro Hashizume, Joel Kolam, Kasis Inape

**Affiliations:** 1 Department of Preventive Medicine, Graduate School of Public Health, Seoul National University, 1 Gwanak-ro, Gwanak-gu, Seoul 08826, Korea; secondmath85@gmail.com; 2 Department of Social and Preventive Medicine, Sungkyunkwan University School of Medicine, 2066 Seobu-ro, Jangan-gu, Suwon, Gyeonggi-do 16419, Korea; kimjh32@skku.edu; 3 Department of Biostatistics and Epidemiology, Graduate School of Public Health, and Institute of Public Health and Environment, Seoul National University, 1 Gwanak-ro, Gwanak-gu, Seoul 08826, Korea; hokim@snu.ac.kr; 4 Graduate School of Comprehensive Human Sciences, University of Tsukuba, 1-1-1 Tennodai, Tsukuba, Ibaraki 305-8577, Japan; honda@taiiku.tsukuba.ac.jp; 5 Department of Preventive Medicine, Dankook University College of Medicine, 119 Dandae-ro, Dongnam-gu, Cheonan, Chungcheongnam-do 31116, Korea; minaha@dku.edu; 6 Institute of Tropical Medicine, Nagasaki University, 1-12-4 Sakamoto Nagasaki 852-8523, Japan; hashizum@nagasaki-u.ac.jp; 7 National Department of Health, P.O. Box 807 Waigani, Port Moresby, National Capital District, Papua New 131, Guinea; joel_kolam@health.gov.pg; 8 National Weather Service, P.O. Box 1240 Boroko, Port Mresby, National Capital District, Papua New 111, Guinea; kinape@pngmet.gov.pg

**Keywords:** climate, seasonality, tropical area, El Niño southern oscillation, Indian Ocean dipole, meta-analysis, vulnerability, Asia-Pacific

## Abstract

This study aimed to assess the association between climate factors and the incidence of childhood pneumonia in Papua New Guinea quantitatively and to evaluate the variability of the effect size according to their geographic properties. The pneumonia incidence in children under five-year and meteorological factors were obtained from six areas, including monthly rainfall and the monthly average daily maximum temperatures during the period from 1997 to 2006 from national health surveillance data. A generalized linear model was applied to measure the effect size of local and regional climate factor. The pooled risk of pneumonia in children per every 10 mm increase of rainfall was 0.24% (95% confidence interval: −0.01%–0.50%), and risk per every 1 °C increase of the monthly mean of the maximum daily temperatures was 4.88% (95% CI: 1.57–8.30). Southern oscillation index and dipole mode index showed an overall negative effect on childhood pneumonia incidence, −0.57% and −4.30%, respectively, and the risk of pneumonia was higher in the dry season than in the rainy season (pooled effect: 12.08%). There was a variability in the relationship between climate factors and pneumonia which is assumed to reflect distribution of the determinants of and vulnerability to pneumonia in the community.

## 1. Introduction

Pneumonia is a major health problem worldwide and is one of the primary causes of death in children under five years of age [1]. Childhood pneumonia is common in the tropical regions, and it accounts for 23% of total child deaths and its case fatality rate has been reported to be up to 2.84% in Papua New Guinea (PNG) [2,3,4].

Pneumonia is an infectious disease transmitted by person-to-person route and is closely associated with climate in its development. Its incidence demonstrates high seasonality, *i.e.*, higher incidence in the winter in the temperate areas and in the rainy season in the tropical areas [5,6,7,8]. Increased opportunity of contact with infected person from increased indoor activity, divergence of the survival and stability of pneumococci in the air, decreased host immunity and behavioral changes of individuals are suggested factors [9].

Prior studies have investigated the relationship between respiratory diseases and various climate factors in the tropical areas. In Malaysia, more rainy days per month and lower monthly mean temperature from November to January were related with higher occurrence of respiratory syncytial virus infection in children [5]. The incidence of hospitalized influenza A pneumonia showed a bimodal seasonal pattern in Thailand [6]. High seasonality of pneumonia in tropical and subtropical areas was interpreted with daytime sunshine hours [9] and increasing ambient temperature in Taiwan [7]. A study in Brazil demonstrated that temperature, rainfall, humidity, and seasonality have various effects on different types of pneumococci [8]. These reports are descriptive in nature, however, which demonstrates a simple correlation [5,6,8] based on their geographic and climatic pattern. Previous studies have not provided sufficient evidence to understand the complex relationship between climate factors and childhood pneumonia incidence in the tropics.

Climate can affect disease pattern in different ways, depending on its basic characteristics, such as geography, land use, demography and socio-cultural status and human behavioral patterns. PNG has a strong diversity in its distribution of ethnicity, sociodemographic status and geography. Therefore, it is expected that effect of climate on specific health outcome such as childhood pneumonia can be different depending on the local determinants of health. Another inquiry that is involved in this area is a climate variability derived from oceanic climate system. Specifically, PNG is located in the Western South Pacific and is heavily affected by the two main oceanic oscillations of the El Niño Southern Oscillation (ENSO) and the Indian Ocean dipole (IOD), which require the inclusion of oceanic effects in any analysis of climate-related events [10,11,12]. Therefore, it provides a unique situation to interpret the complex interrelationship between regional and local climate system on the background of diverse geography and residential settings. In the present study, we quantitated the effect of local and oceanic climate variability on the incidence of childhood pneumonia in the six provinces of PNG and evaluated the variability of the effect size according to their geographic properties.

## 2. Materials and Methods

### 2.1. Study Area

PNG lies in the eastern half of New Guinea island; the second biggest island on the globe, which is comprised of a big land mass with a high mountainous range; and neighboring islands in the western part of South Pacific. Upon closer inspection, PNG has several distinct climate systems, defined by its topography and relative location to the equator and oceans. The northern coastal region faces the equator and experiences heavy rainfall within its rainforest area. The southern coastal region has relatively scarce rainfall and can mostly be defined as a savanna climate. A high mountain range characterizes the highland region, with elevations mostly above 1200 meters and reaching up to 4509 meters at Mt. Wilhelm. Its climate is milder, with moderate amounts of rainfall year-round. The island region has a climate typical of the Western Pacific Oceanic. PNG also has a rich diversity in its ecosystem and ethnicity. The strong influence of the traditional local lifestyle is partly responsible for the limited transport system and the underdevelopment of the national economy with great regional variation.

**Figure 1:**
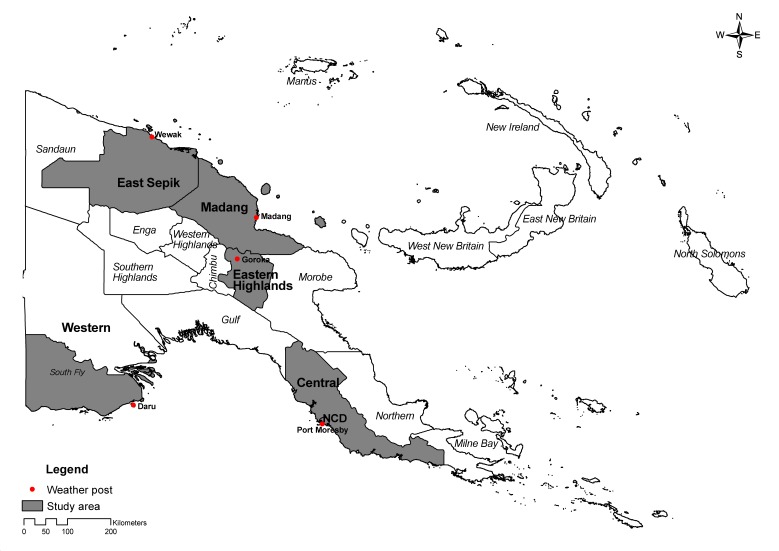
Map of the study areas in Papua New Guinea.

For this study, we selected at least one location from each region, except the island region: Port Moresby (Central Province), and Daru (southern part of the Western Province) represented the southern coastal region. Goroka was chosen for the highland region, and Madang and Wewak (East Sepik Province) represented the northern coastal region (Figure 1).

### 2.2. Data Sources

The monthly number of pneumonia cases in patients under the age of five was collected from the National Health Information System (NHIS) of the National Department of Health, PNG [13]. The NHIS is a nationwide surveillance system of health status and morbidity, which encompasses all health care units. Monthly reports of all the cases of the disease are collected at the province level (PHIS), and then the statistics are reported to the National Department of Health (NDOH) in electronic form. Only the number of cases stratified by age group and gender are assembled in contingency tables; therefore, no personal information is included [13]. Local climate data were obtained from the National Weather Service from 1997 to 2006 over five areas (Port Moresby and Central province, Daru, Eastern Highland Province, Madang Province, and East Sepik Province) representing the southern coastal, highland and northern coastal regions. For the Central Province and Port Moresby, the same local climate data were used. To increase the power of the monthly data, missing values were imputed based on the monthly mean of climate variables over the study period. Because PNG came under the strong influence of the El Niño event in 1997, any missing values for the Eastern Highland Province’s rainfall data for that specific year were instead predicted via a multiple linear regression using other local and global meteorological data as independent variables. The strength of the ENSO was measured with the Southern Oscillation Index (SOI), and that of the IOD was assessed using the Dipole Mode Index (DMI). The SOI is a standardized index based on the observed sea level pressure differences between Tahiti, French Polynesia, and Darwin, Australia, measured by the Bureau of Meteorology, Australia; DMI is defined as the difference between sea surface temperature in the western (50°E to 70°E and 10°S to 10°N) and eastern (90°E to 110°E and 10°S to 0°S) equatorial Indian Ocean [14,15].

### 2.3. Variables in This Study

Monthly pneumonia cases in patients under the age of five years were the dependent variable in this study. Independent variables were yearly and seasonal climate factors such as the dry season (from April to September) and the rainy season (from October to March), and local climate information including monthly rainfall (mm), and monthly mean of daily maximum temperatures (°C). SOI and DMI served as indicators of oceanic climate system information. The time period included in our analysis ranged from 1997 to 2006.

### 2.4. Statistical Analysis

First, we analyzed the descriptive statistics of monthly childhood pneumonia cases, geographic and climate variables in the six provinces. Then, we plotted time-series plots of climate variables and performed a univariate analysis between childhood pneumonia cases and climate and seasonal variables using Spearman correlation coefficients. After confirming the linearity of all climate and seasonal variables using non-parametric multiple analysis via a generalized additive model (GAM), and multi-collinearity of the model using variance inflation factors (VIF), we conducted a parametric multivariate analysis using a generalized linear model (GLM). VIF is widely used measure of the multi-collinearity of the independent variables with other independent variables in GLM [16]. Finally, we carried out a meta-analysis on each variable in the six areas after checking the heterogeneity of results in research.

### 2.5. GAM and GLM Analysis

Because disease incidence or mortality is commonly assumed to follow a Poisson process, GLM should be considered rather than a conventional linear regression, whose distribution family is a Poisson distribution and link function is a log function [17,18]. At the same time, linearity should be checked before applying a GLM because there can be a non-linear relationship between climate change and disease risk. PNG is one of the tropical areas that have a narrow range of temperature variation; linearity for the monthly mean of max temperature can be assumed, but linearity of other climate variables cannot. Therefore, we employed a GAM as a non-parametric estimation for those other variables to examine the linearity between climate variables and the log(RR) [19]. We utilized penalized, thin-plate regression splines as a smoothing function of the GAM. Thin-plate regression splines is useful if we need to check that a smooth term could be replaced by a linear term and it have the advantage that term with a modification is shrunk to zero for high enough smoothing parameter [20]. The autocorrelation function (ACF) of disease risk in each province were checked to find the significant lags for all province in GLM model. After confirming the comparability of a linear assumption in GAM, we performed an analysis applying a GLM as a parametric estimation.

Equations for the GAM (Equation (1)) and the GLM (Equation (2)) are shown below:

*Log(μ_i_) = β_0_ + s(rainfall_i_) + s(tamx_i_) + s(SOI_i_) + s(DMI_i_) + factor(year) + I(dry season) + ε_i_* (1)

*Log(μ_i_) = β_0_ + β_1_∙ rainfall_i_ + β_2_∙ tamx_i_ + β_3_∙ SOI_i_ + β_4_∙ DMI_i_ + factor(year) + I(dry season) + ε_i_* (2)

(μ*_1_*: expected monthly case count in *i*th observation, *ß_0_*: intercept, *s*: smoothing function, *factor(year)*: indicator variables for the years (1997:2006), *I(dry season)*: 1—dry season, 0—rainy season, ε*_i_*: error term).

### 2.6. Meta-Analysis in the Six Areas

After estimating the effects of climate variables on childhood pneumonia incidence in each of the six areas, a meta-analysis was performed to obtain the overall risk that combined the results from the different regions. This result can be assumed as a pooled effect of climate and seasonal changes on childhood pneumonia incidence in PNG. In applying the meta-analysis, the authors assumed that the effect size of climate factor on disease could be different among the six areas because of the high variability of geography and sociocultural status, which was confirmed by testing the heterogeneity of all the variables. Therefore, we applied a restricted maximum likelihood (REML) estimation method (one of the random effect models), rather than a fixed effect model, to estimate the pooled effect [21]. All tests were done at a significance level of 0.05, and all the data steps and statistical analyses were performed using the R 2.15.1; package mgcv for GAMs and package metaphor for the meta-analysis in R.

## 3. Results

### 3.1. General Characteristics of the Study Areas

Descriptive statistics of childhood pneumonia cases and geographic and climate variables in the six areas are shown in Table 1. The population density ranged from 2.37 person/km^2^ in Daru to 2468 person/km^2^ in Port Moresby. The spectrum of the population size under five years of age was from 20,166 in Daru to 90,391 in Port Moresby. Childhood pneumonia incidence under five years was the highest in the Eastern Highland Province (39.1 ± 14.1 per 1000 children) and the lowest in Daru (9.4 ± 3.2 per 1000 children).

**Figure 2:**
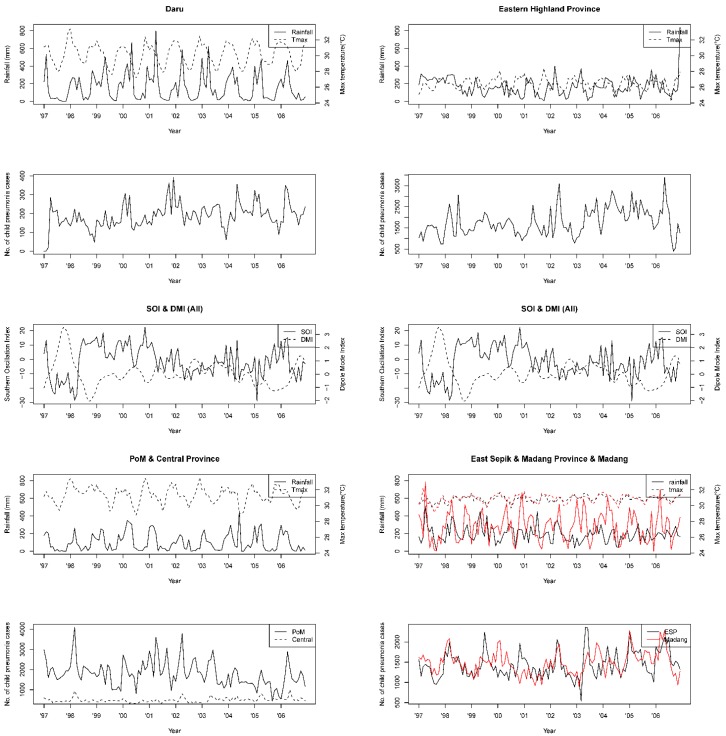
Monthly time-series: childhood pneumonia cases in children aged five years; rainfall, max temperature (°C), dipole mode index (DMI), southern oscillation index (SOI), from 1997 to 2006.

The monthly means of daily maximum temperatures were similar for all of the areas except for the Eastern Highland Province, which is located in the highlands. Additionally, there was little annual temperature variation among all areas, with a mean difference of 2.7 °C. In contrast, both rainfall and seasonality showed a large disparity among the regions. The northern coastal area, Madang Province and East Sepik Province had higher rainfall, and Madang Province’s amount of rainfall showed no discrete difference between the rainy and the dry seasons. Although located in the same northern coastal region, a significant inconsistency was detected between the Madang Province and the East Sepik Province in rainfall amount due to the topological influence of a high mountainous district in the Madang Province. In the southern coastal area, a rainfall discrepancy between the rainy and dry seasons was distinctive. The Western Province, Daru, had a far higher rainfall in the rainy season, and Port Moresby and the Central Province, both representing the savanna climate, showed a low mean rainfall and very little precipitation in the dry season. The highland region in the Eastern Highland Province had low rainfall and a relatively small difference between its seasons. The means and standard deviations of SOI and DMI during the study period were −0.94 ± 10.7 and 0.06 ± 0.99, and 1997–1998 was the heaviest El Niño period with a minimum monthly SOI value of −28.5 (Table 1, Figure 2).

### 3.2. Seasonal Variation in Variables and Correlations of Childhood Pneumonia Cases and Climate Variables

The seasonality of childhood pneumonia cases and climate variables of the six areas are described in Figure 2. Both cases and climate variables had a high seasonality pattern, and when calculating Spearman correlation coefficients between the number of cases and climate variables, we detected a significant negative correlation between cases and SOI in the Central Province (−0.13) and also between cases and maximum temperature (−0.335) and DMI (−0.146) in the Eastern Highland Province. A significant positive correlation was observed between cases and dry season effect (0.353). In the East Sepik Province, only DMI had a significant correlation with the number of cases (−0.221). In the Madang Province, rainfall (0.188) and DMI (−0.223) both had a significant correlation with the number of pneumonia cases (Table 2).

**Table 1:** Descriptive statistics of the six areas in Papua New Guinea between 1997–2006 (mean ± SD).

Variables	Season	Daru (Western Province)	Port Moresby	Central Province	Eastern Highland Province	East Sepik Province	Madang Province
Population density (person/km^2^)		2.4 ± 0.3	2468.0 ± 373.0	5.7 ± 0.4	30.4 ± 1.7	6.5 ± 0.4	51.3 ± 4.8
Population under five years old^†^		20,166	90,391	27,989	49,365	51,931	59,310
Pneumonia under five years old (/month)	Total	188.3 ± 67.6	1809.9 ± 637.8	491.3 ± 119.4	1780.3 ± 651.0	1434.2 ± 323.8	1481.4 ± 287.5
	Rainy	181.2 ± 79.4	1764.2 ± 655.7	487.0 ± 101.6	1567.1 ± 570.8 *	1381.5 ± 300.1	1491.3 ± 318.6
	Dry	195.4 ± 52.4	1855.6 ± 616.0	495.7 ± 134.7	1993.6 ± 656.4 *	1487.0 ± 337.7	1471.6 ± 252.2
Incidence (/month, /1000 persons)	Total	9.4 ± 3.2	25.6 ± 10.3	19.6 ± 4.6	39.1 ± 14.0	30.8 ± 6.7	28.9 ± 5.7
	Rainy	9.0 ± 3.7	25.2 ± 11.3	19.5 ± 4.2	34.4 ± 12.3 *	29.6 ± 6.2	29.2 ± 6.5
	Dry	9.9 ± 2.6	26.0 ± 9.1	19.7 ± 5.0	43.8 ± 14.1 *	31.9 ± 7.0	28.7 ± 4.9
Rainfall (mm/month)	Total	159.7 ± 161.6	100.1 ± 101.6	100.1 ± 101.6	167.6 ± 106.6	193.3 ± 89.1	281.6 ± 166.3
	Rainy	180.8 ± 132.1	135.0 ± 97.0 *	135.0 ± 97.0 *	196.2 ± 119.3 *	171.1 ± 68.3 *	323.7 ± 138.9 *
	Dry	138.6 ± 184.1	65.3 ± 93.8 *	65.3 ± 93.8 *	139.0 ± 82.7 *	215.5 ± 101.2 *	239.6 ± 180.3 *
Mean of daily maximum temperature (°C) ^‡^	Total	30.1 ± 1.4	31.3 ± 1.1	31.3 ± 1.1	26.4 ± 0.7	30.8 ± 0.4	30.9 ± 0.5
	Rainy	31.3 ± 0.7 *	32.1 ± 0.7 *	32.1 ± 0.7 *	26.8 ± 0.6 *	30.9 ± 0.4 *	31.2 ± 0.4 *
	Dry	29.0 ± 0.9 *	30.6 ± 0.8 *	30.6 ± 0.8 *	26.0 ± 0.6 *	30.7 ± 0.4 *	30.6 ± 0.5 *
SOI	Total	−1.61 ± 10.90					
	Rainy	0.43 ± 11.32 *					
	Dry	−3.64 ± 10.06 *					
DMI	Total	0.06 ± 0.99					
	Rainy	−0.01 ± 1.10					
	Dry	0.12 ± 0.86					
Malnutrition (persons)	Total	17.3 ± 56.5	44.5 ± 67.3	27.4 ± 23.3	53.0 ± 37.4	54.7 ± 39.5	39.1 ± 26.9
	Rainy	11.4 ± 12.3	39.9 ± 29.6	27.8 ± 22.9	54.4 ± 43.7	45.3 ± 34.3 *	36.1 ± 23.7
	Dry	23.2 ± 78.5	49.1 ± 90.2	27.0 ± 23.7	51.6 ± 29.6	64.2 ± 42.0 *	42.1 ± 29.4

SD: Standard deviation, SOI: Southern oscillation index, DMI: Diploe mode index, ^†^ As of 2010, ^‡^ Monthly mean of daily maximum temperature, *: *p*-value <0.05.

### 3.3. Fitness of the Statistical Models (Degree of Freedoms, Autocorrelation and Collinearity).

When confirming the linearity of climate variables, a sensitivity analysis was conducted by comparing the results with adjusting for the climate variables and by using different degrees of freedom for rainfall (1, 2, 3, 4 and 5 df), SOI (1, 2, 3, 4 and 5 df), DMI (4, 5, 6, 7 and 8 df), max temperature (1, 2, 3,4 and 5 df). The results are shown in Table S1. The ACF of the GLM model using monthly data in each province did not show a significant autocorrelation (Figure S1). These results suggested that research areas in statistical modeling did not have distinctive heterogeneities between childhood pneumonia and monthly climate factors. Tests for collinearity with the VIF indicated that all of climate related variables are acceptable with the weak multicollinearity. Values of VIF are presented in Table S2 as the measure of collinearity in the model.

**Table 2:** Spearman correlation coefficients for independent variables of climate, malnutrition cases and childhood pneumonia cases under five years of age in six regions in Papua New Guinea between 1997–2006.

Variables	Season	Daru (Western Province)	Port Moresby	Central Province	Eastern Highland Province	East Sepik Province	Madang Province
Rainfall (mm/month)	Total	−0.001	0.107	0.107	−0.028	0.126	0.188 *
	Rainy	−0.031	0.058	0.063	0.138	0.144	0.080
	Dry	0.099	0.297 *	0.232	−0.013	0.045	0.321 *
SOI	Total	−0.164	−0.048	−0.130	−0.116	−0.060	−0.077
	Rainy	−0.216	−0.045	−0.047	−0.032	0.055	−0.080
	Dry	−0.085	−0.042	−0.215	−0.009	−0.095	−0.056
DMI	Total	0.051	0.133	−0.184 *	−0.146	−0.221 *	−0.223 *
	Rainy	0.148	0.215	−0.144	−0.110	−0.173	−0.139
	Dry	−0.026	0.065	−0.246	−0.379 *	−0.334 *	−0.338 *
Dry season	Total	0.113	0.087	−0.003	0.353 *	0.140	−0.001
Malnutrition (cases)	Total	0.364 *	0.306 *	0.081	0.473 *	0.324 *	0.205 *
	Rainy	0.471 *	0.370 *	−0.100	0.571 *	0.155	0.149
	Dry	0.263 *	0.246	0.265 *	0.398 *	0.432 *	0.227

SOI: Southern oscillation index, DMI: Diploe mode index; ^†^: As of 2010, ^‡^: Monthly mean of daily maximum temperature; *: *p*-value < 0.05.

### 3.4. Generalized Additive Model and Generalized Linear Model

**Figure 3:**
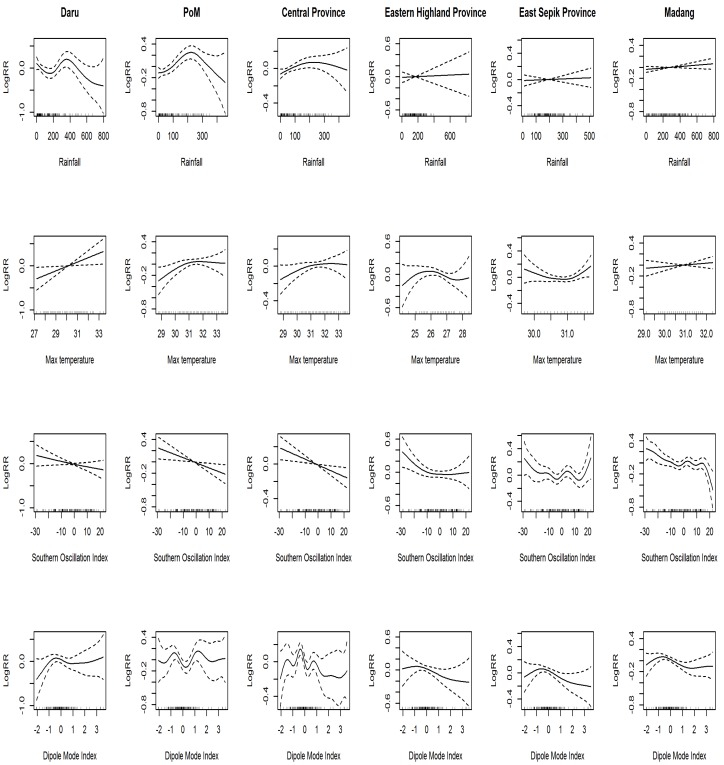
Log(RR)s of independent variables using a generalized additive model in six geographic areas in Papua New Guinea.

**Figure 4:**
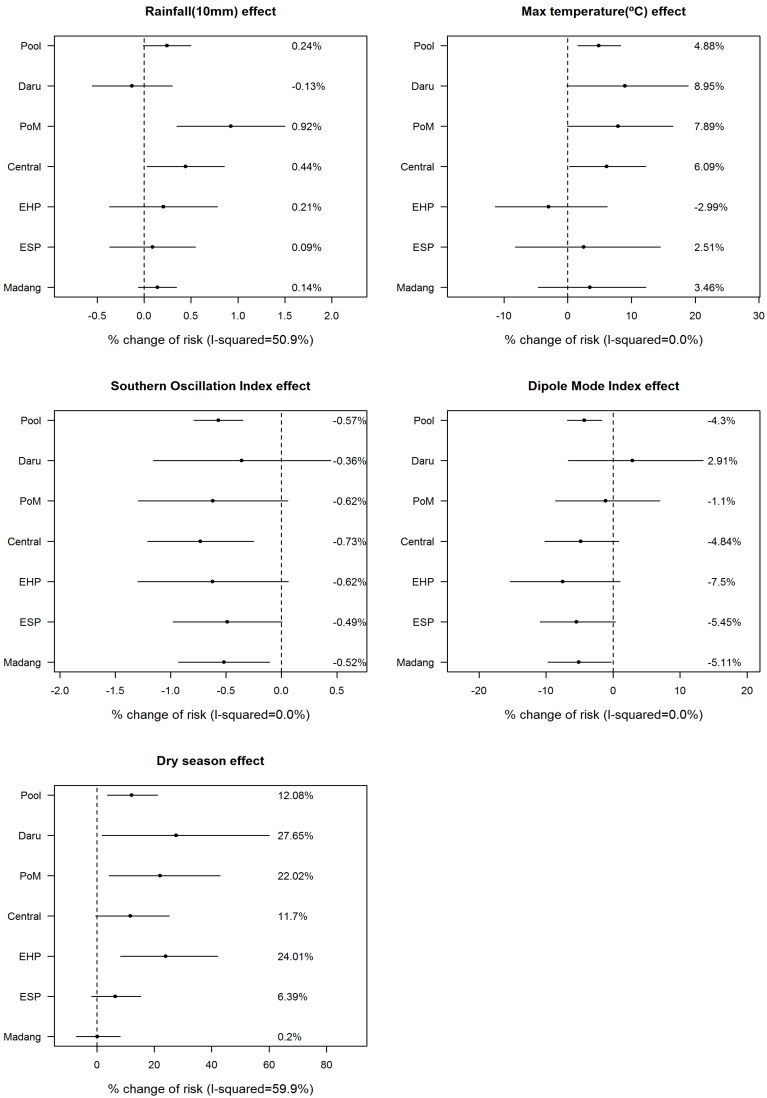
Percent change of risk and meta-analysis in six regions in Papua New Guinea PoM: Port Moresby, EHP: Eastern Highland Province, ESP: East Sepik Province.

First, using a GAM, we checked the linearity between childhood pneumonia risk in log (RR) and climate variables except for maximum temperature (Figure 3). Based upon the results, the linearity assumption was concluded to be acceptable; therefore, we quantitatively estimated the risk using a GLM (Table 3). Figure 4 describes the percent changes in risk in the six areas and the pooled effect via meta-analysis using an REML algorithm. The risk of pneumonia was increased by −0.13%, 0.92%, 0.44%, 0.21%, 0.9%, and 0.14% for every 10-mm increase in rainfall for Daru, Port Moresby, Central Province, Eastern Highland Province, East Sepik Province, and Madang Province, respectively; the pooled effect percent change in risk was 0.24 (95% CI: −0.01:0.5). The risk of pneumonia rose by 8.95%, 7.89%, 6.09%, −2.99%, 2.51%, and 3.46% per 1 °C increase of max temperature, respectively. The pooled percent change in risk appeared to be 4.88% (95% CI: 1.57:8.3). For SOI, RR varied by −0.36%, −0.62%, −0.73%, −0.62%, −0.49%, and −0.52% per 1-unit increase of SOI, respectively; the overall RR percent change in risk was obtained as −0.57 (95% CI: −0.79:−0.35). For DMI, the percent change in risk was 2.91, −1.1, −4.84, −7.5, −5.45, and −5.11 per 1-unit increase of DMI; the pooled effect was −4.3% (95%CI: −6.83:−1.68). Finally, in the dry season, risks were 27.65%, 22.02%, 11.7%, 24.01%, 6.39%, and 0.2% higher than the rainy season for each area in the same order as above, and the overall RR percent change was 12.08% (95% CI: 3.73:21.11).

**Table 3:** Relative risk for independent variables of climate for childhood pneumonia using generalized linear model in six areas in Papua New Guinea between 1997 and 2006.

Climate Factors	RR (95% CI)					
	Daru (Western Province)	Port Moresby	Central Province	Eastern Highland Province	East Sepik Province	Madang Province
Rainfall (10 mm/month)	0.999	1.009 *	1.004 *	1.002	1.001	1.001
	(0.994–1.003)	(1.003–1.015)	(1.000–1.009)	(0.996–1.008)	(0.996–1.005)	(0.999–1.003)
Mean of daily maximum temperature (°C) ^‡^	1.090	1.079	1.061 *	0.970	1.025	1.035
	(0.998–1.189)	(0.999–1.165)	(1.003–1.122)	(0.886–1.062)	(0.918–1.145)	(0.953–1.122)
SOI	0.996	0.994	0.993 *	0.990	0.995	0.995 *
	(0.988–1.004)	(0.987–1.001)	(0.988-0.997)	(0.987–1.001)	(0.990–1.000)	(0.991-.999)
DMI	1.029	0.989	0.952	0.925	0.946	0.949*
	(0.933–1.134)	(0.914–1.070)	(0.898–1.008)	(0.846–1.010)	(0.891–1.003)	(0.903-.997)
Season^†^	1.276 *	1.220 *	1.117	1.240 *	1.064	1.002
	(1.018–1.599)	(1.041–1.428)	(0.995–1.253)	(1.083–1.420)	(0.982–1.153)	(0.928–1.082)

RR: Relative risk, CI: Confidence interval, SOI: Southern oscillation index, DMI: Dipole mode index; ^†^: Rainy season as a reference, ^‡^: Monthly mean of daily maximum temperature. *: *p*-value <0.05.

## 4. Discussion

In this study, we demonstrated the effect of local and global climate factors on childhood pneumonia in a developing tropical country. Effects of climate factors varied depending on the region. The childhood pneumonia risk generally showed a positive association with rainfall, maximum temperature and the dry season, and a negative association with SOI and DMI in the six areas of PNG.

In comparison with previous studies, a positive association between rainfall and childhood pneumonia incidence supported the existing theory that childhood pneumonia cases increase in the rainy season, and also was consistent with the results of Chen *et al.* [5,9]. The positive effect of maximum temperature was in agreement with the results of Lin et al [7], but was opposite to those of Chan et al [5]. At the same time, childhood pneumonia incidence generally increased in the dry season in our study, which contradicts our results regarding the positive association with rainfall and also contradicts past research on increased pneumonia risk in the rainy season [9]. This finding suggested that there may be a dry season effect that excludes the rainfall effect. A possible explanation may be that malnutrition worsens with food shortages during the dry season. In the highland area of PNG, children are vulnerable to the effect of drought, which is reflected in the drop of birth weight immediately after the El Niño event [22]. The Spearman correlation coefficient of childhood pneumonia cases and malnutrition cases from our data was 0.3 (95% CI: 0.24–0.37), and there were significantly more malnutrition cases in the dry season than in the rainy season (difference = 7.03, *p*-value = 0.04). This finding was not in agreement with a previous study that reported that food shortages occur more frequently in the rainy season, because the rainy season is a pre-harvest period in Africa and Asia [9].

SOI and DMI, which were used as global oceanic effect measures, had a negative association with childhood pneumonia risk, indicating that there was a positive association between the phenomenon of El Niño and childhood pneumonia risk. The relationship of oceanic sea surface temperature indexes with the health effects have been demonstrated in vector-borne diseases including dengue and malaria [23,24] and fecal-oral infectious diseases, such as cholera. However, the relationship of these indexes with respiratory diseases have rarely been reported. Oceanic sea surface temperature indexes, such as SOI and DMI, can affect human health primarily through its effect on the local climate variability [14,25]. They have strong impact on the ecology of vectors and crop failures resulting in a prolonged drought leading to crop failure and malnutrition [22]. Aggravation of air pollution from forest fire is another factors related with increased respiratory disease [26]. In contrast to the wide variation of the effect of local climate, both ENSO and IOD had a more consistent effect across the localities in PNG. This result also suggests a significant influence of, not only ENSO, but also IOD on the climate and its health effect in PNG, in spite of its location is out of the scope of the Indian Ocean. During the negative IOD, sea surface temperature of the designated area around the Sumatra Island goes up by definition, but there is few published evidence of increased sea surface temperature nearby PNG.

We found there are regional differences in effect size. However, directions of which mostly coincided. Amount of monthly rainfall had a positive relationship with childhood pneumonia in the Central Province and Port Moresby. In the GAM analysis, positive relationship was visible only until 200 mm then it turned into a negative relationship. Effect of rainfall on pneumonia had a moderate level of heterogeneity (I^2^ = 50.9%), which may be related with a different dose-response relationship between regions. Daily maximum temperature was positively related with childhood pneumonia in the southern coastal regions, while this effect was not evident in the other regions. SOI had a general influence on the risk of pneumonia across the nation except in Daru, located at the southwestern region of the country, while DMI had a limited influence, significant only in Madang province. It is paradoxical to find an impact of dry season on the pneumonia, which was observed across the southern coastal regions to highland region, but not on the northern coastal region. The regions which showed a worsening of pneumonia during the dry season had a bigger difference in the rainfall and temperature, *i.e.*, it was drier and hotter during the dry season. The Eastern Highland Province, located in the highland region, has a relatively lower temperature than other areas, which supports the presumption that topographical features influence the effect of temperature on pneumonia risk. The Madang Province is a region of high precipitation where the mean amount of rainfall can be 239 mm even during the dry season, much higher than the mean of the other areas in the rainy season (160 mm). Therefore, the Madang Province had a relatively lower dry season effect. Another possible explanation might be that the relatively high socioeconomic status in Madang Province might dilute the effects of fluctuations in the environmental system in the dry season. In fact, in Madang Province, there was no significant difference in malnutrition cases during the dry and the rainy season (difference = 6.05, *p*-value = 0.22).

Limitations of our study include the unavailability and inaccuracy of childhood pneumonia case data. PNG is a country with an insufficient number of doctors; pneumonia is frequently diagnosed by non-physicians, so it is unlikely that all pneumonia cases are actually reported. Therefore, distribution of childhood pneumonia by its etiology was not available, which may have obscured the actual relationship compared with that based on cause-specific pneumonia. Further, the local climate data only include rainfall and temperature information, and there were some missing monthly reports. In our study, the missing information was replaced with the mean value of that month. The Eastern Highland Province had missing values for rainfall in 1997, which were imputed using other climate variables as a reference. Possible inaccuracies due to this imputation might have influenced the results of our study. Using monthly data due to the lack of available daily data may have reduced power of our study because the sample number became smaller, and any potential lag effect of climate variables could not be examined. Data with lower temporal resolution, such as weekly or monthly data instead of daily data, may obscure the time-dependent relationship between climate factors and health outcome, especially in acute diseases. A more thorough study could be conducted with more accurate data. In data analysis, multi-collinearity of the climate variables may be problematic. Simplifying variables with the principal component analysis and other approaches may be one possible solution. The misdiagnosis of pneumonia would potentially affect the results of this study, but only if over- (or under-) diagnosis was associated with the climatic factors of interest, and there is no evidence for this. This study is an ecologic in its nature, which are liable to the ecologic fallacy. Time dependent variability of health outcome depending on the climate factors may be misleading, resulting from false relationship.

Despite the aforementioned concerns, our study was useful in that we successfully demonstrated the association between childhood pneumonia risk and local/oceanic climate variability and estimated the effect size of climate and seasonality. We find the different direction of effect size of a specific climate factor may reflect the different distribution of the basic mechanism of the pneumonia development in the local area. Thus, through the pattern of response to the specific climate factors on the pneumonia risk, we may accept or reject a specific determinants of pneumonia development in the area. For example, strong effect of dry season on the risk of pneumonia development in most of the areas may suggest that explanation based on the higher opportunity of transmission during the wet season is not a plausible explanation in this case. Rather, heightened susceptibility to pneumonia related with food shortage during dry season may be a more plausible explanation. Therefore, we believe that heterogeneity of direction and size of the response to the specific climate factors in a specific area indicates the different distribution of the main determinants of the risk of the pneumonia development in the area.

In addition, we demonstrated that oceanic climate variability, such as ENSO and IOD, as well as local climate changes, were associated with childhood pneumonia incidence, and that climate effects can be different according to differing features of an area. We find the time-series data analysis based on the long-term surveillance data and application of meta-analysis can provide a potent insight on the impact and mechanism of climate on the health effect while taken the local as well as regional climate variability into account. Surveillance data is available in most of the countries and regions, and in spite of its limitations in terms of validity, it could provide a quite consistent and robust data to analyze the chronological relationship between long-term climate and its health effects. These findings will serve as a baseline reference for future studies in other tropical regions, and will help identify the effects of climate variability on specific health risk.

## 5. Conclusions

We identified the relationships between local and global climate factors and the risk of childhood pneumonia in Papua New Guinea. However, the effect size and direction can be widely variable according to geographic features and local climate variables. It suggests that local health determinants may work as an effect modifier on the relationship of climate factors on the development of pneumonia.

## Supplementary Materials

The following are available online at www.mdpi.com/1660-4601/13/2/213/s1, Table S1: Percent change (95% C.I.) of childhood pneumonia cases and each variables with different degree of freedom (df) in GAM model, Table S2: Values of the variance inflation factor (VIF) as a measure of the collinearity observed in variables from each areas. Figure S1: Autocorrelation of the GLM model using monthly data in each province.

## Abbreviations

CI: confidence interval
DMI: dipole mode index
ENSO: El Niño southern oscillation
GAM: generalized additive model
GLM: generalized linear model
IOD: Indian Ocean dipole
NDOH: National Department of Health, Papua New Guinea
NHIS: National Health Information System (Papua New Guinea)
PNG: Papua New Guinea
RR: relative risk
SOI: southern oscillation index
ACF: autocorrelation function
