# Supplementary material for: Effect of Climate Factors on the Childhood Pneumonia in Papua New Guinea: A Time-Series Analysis
Source: Int J Environ Res Public Health. 2016 Feb 15;13(2):213. doi: 10.3390/ijerph13020213 (PMC4772233; doi:10.3390/ijerph13020213)
Supplement: Supplementary file 1 [file ijerph-13-00213-s001.pdf]

Supplementary Materials: Effect of Climate Factors on the Childhood Pneumonia in Papua New Guinea: A Time-Series Analysis

Jinseob Kim , Jong-Hun Kim , Hae-Kwan Cheong *, Ho Kim , Yasushi Honda , Mina Ha , Masahiro Hashizume , Joel Kolam and Kasis Inape

Table S1. Percent change (95% C.I.) of childhood pneumonia cases and each variables with different degree of freedom (df) in GAM model

Variables	df	Rainfall (per 10 mm)	SOI (per 1 Unit)	DMI (per 1 Unit)	Max temperature (per 1 °C)
Rainfall	df = 1		−0.690 (−1.173, −0.204)	−4.323 (−9.579, 1.237)	4.582 (−1.036, 10.519)
	df = 2		−0.659 (−1.138, −0.178)	−3.306 (−8.657, 2.358)	3.303 (−2.282, 9.208)
	df = 3		−0.716 (−1.209, −0.211)	−3.538 (−8.930, 2.174)	4.154 (−1.589, 10.233)
	df = 4		−0.768 (−1.279, −0.255)	−3.499 (−8.939, 2.265)	3.744 (−1.994, 9.818)
	df = 5		−0.766 (−1.278, −0.251)	−3.277 (−8.767, 2.543)	3.715 (−2.071, 9.842)
SOI	df = 1	0.274 (−0.153, 0.702)		−3.538 (−8.930, 2.174)	4.154 (−1.589, 10.233)
	df = 2	0.277 (−0.160, 0.716)		−3.484 (−8.915, 2.270)	4.130 (−1.786, 10.402)
	df = 3	0.283 (−0.158, 0.726)		−3.487 (−8.944, 2.298)	4.370 (−1.610, 10.712)
	df = 4	0.278 (−0.166, 0.725)		−3.382 (−8.864, 2.429)	4.606 (−1.441, 11.024)
	df = 5	0.268 (−0.182, 0.719)		−3.653 (−9.226, 2.263)	4.197 (−1.992, 10.776)
DMI	df = 4	0.233 (−0.186, 0.654)	−0.732 (−1.230, −0.232)		4.706 (−1.089, 10.841)
	df = 5	0.330 (−0.091, 0.752)	−0.732 (−1.219, −0.243)		4.440 (−1.225, 10.429)
	df = 6	0.274 (−0.153, 0.702)	−0.716 (−1.209, −0.221)		4.154 (−1.589, 10.233)
	df = 7	0.297 (−0.121, 0.716)	−0.646 (−1.130, −0.160)		3.266 (−2.323, 9.175)
	df = 8	0.319 (−0.098, 0.739)	−0.638 (−1.119, −0.154)		3.079 (−2.481, 8.955)
Max temperature	df = 1	0.355 (−0.069, 0.780)	−0.669 (−1.166, −0.171)	−4.006 (−9.435, 1.748)	
	df = 2	0.274 (−0.153, 0.702)	−0.716 (−1.209, −0.221)	−3.538 (−8.930, 2.174)	
	df = 3	0.282 (−0.148, 0.713)	−0.714 (−1.209, −0.217)	−3.795 (−9.266, 2.006)	
	df = 4	0.333 (−0.098, 0.766)	−0.652 (−1.149, −0.151)	−3.598 (−9.122, 2.261)	
	df = 5	0.354 (−0.072, 0.782)	−0.584 (−1.083, −0.083)	−2.807 (−8.455, 3.189)	

Optimal df in our fitted model: Rainfall (3.5 df), SOI (1.0 df), DMI (6.3 df), Max temperature (2.1 df).

Table S2. Values of the variance inflation factor (VIF) as a measure of the collinearity observed in variables from each areas

Province	Variables	GVIF	*Df*	$GVIF^{\frac{1}{2Df}}$	Province	Variables	GVIF	*Df*	$GVIF^{\frac{1}{2Df}}$
Daru	Rainfall	1.31	1	1.14	Eastern Highland	Rainfall	1.28	1	1.13
	Max temperature	3.87	1	1.97		Max temperature	1.49	1	1.22
	SOI	1.92	1	1.38		SOI	1.85	1	1.36
	DMI	2.18	1	1.48		DMI	2.21	1	1.49
	Factor(year)	2.64	9	1.06		Factor(year)	2.72	9	1.06
	Factor(season)	3.38	1	1.84		Factor(season)	1.59	1	1.26
PoM	Rainfall	1.30	1	1.14	East Sepik	Rainfall	1.18	1	1.09
	Max temperature	2.39	1	1.54		Max temperature	1.38	1	1.17
	SOI	2.14	1	1.46		SOI	2.08	1	1.44
	DMI	2.21	1	1.48		DMI	2.28	1	1.51
	Factor(year)	2.66	9	1.06		Factor(year)	2.88	9	1.06
	Factor(season)	2.29	1	1.51		Factor(season)	1.15	1	1.07
Central	Rainfall	1.18	1	1.09	Madang	Rainfall	1.20	1	1.10
	Max temperature	1.38	1	1.17		Max temperature	1.76	1	1.33
	SOI	2.08	1	1.44		SOI	2.13	1	1.46
	DMI	2.28	1	1.51		DMI	2.45	1	1.57
	Factor(year)	2.88	9	1.06		Factor(year)	2.68	9	1.06
	Factor(season)	1.15	1	1.07		Factor(season)	1.50	1	1.23

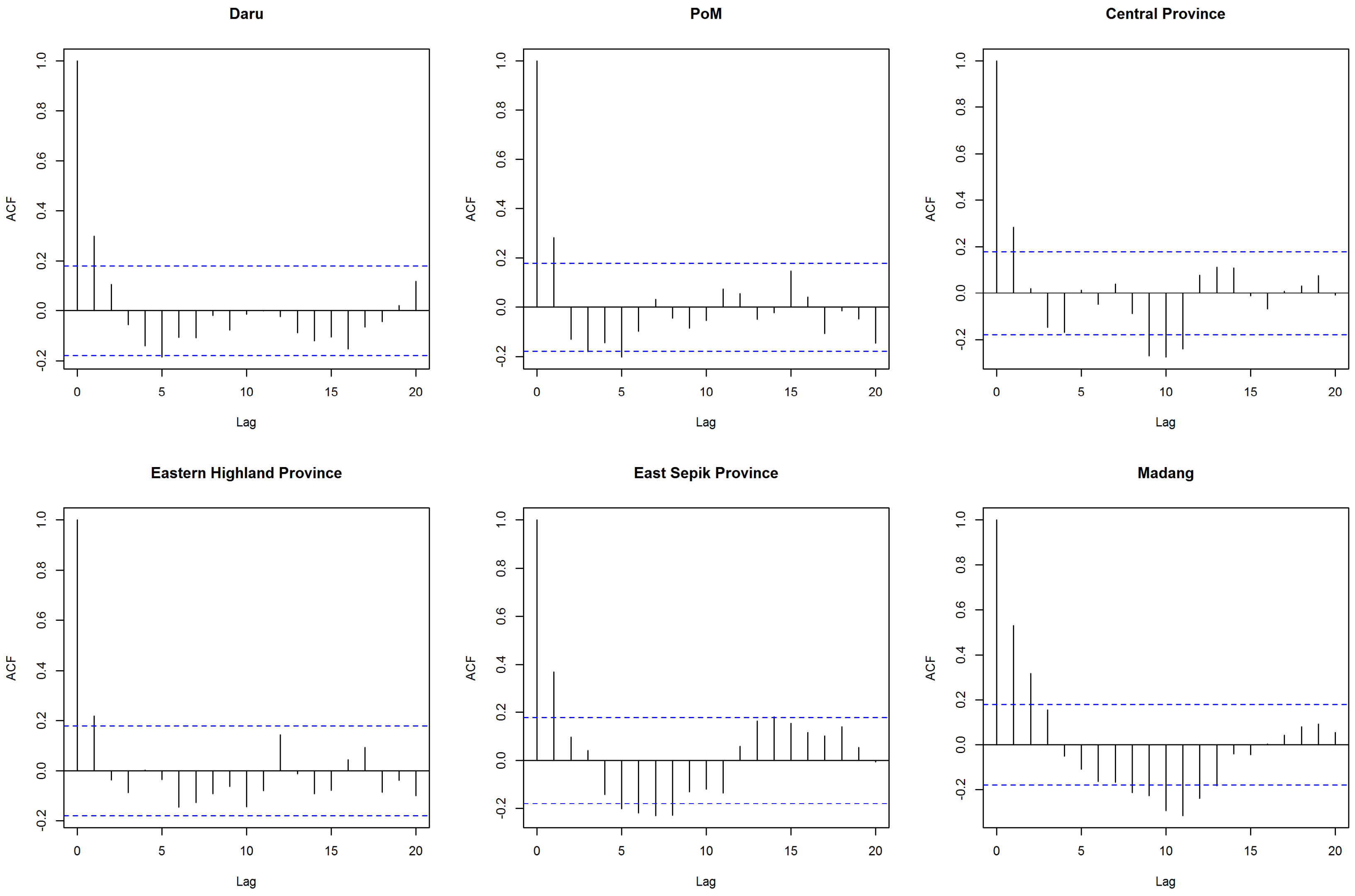

Figure S1. Autocorrelation of the GLM model using monthly data in each province.

© 2016 by the authors; licensee MDPI, Basel, Switzerland. This article is an open access article distributed under the terms and conditions of the Creative Commons by Attribution (CC-BY) license (http://creativecommons.org/licenses/by/4.0/).
